# Nucleus accumbens deep brain stimulation in adult patients suffering from severe and enduring anorexia nervosa (STIMARS): protocol for a pilot study

**DOI:** 10.3389/fpsyt.2025.1554346

**Published:** 2025-03-20

**Authors:** Philibert Duriez, Giorgia Antonia Simboli, Philippe Domenech, Anne Buot, Casey Halpern, Marie Fadigas, Yann Mongin, Aurore Guy-Rubin, Romain Carron, Catherine Oppenheim, Philip Gorwood, Johan Pallud, Marc Zanello

**Affiliations:** ^1^ Groupe Hospitalier Universitaire (GHU) Paris Psychiatrie et Neurosciences, Clinique des Maladies Mentales et de l’Encéphale, Hôpital Sainte Anne, Paris, France; ^2^ Laboratoire de Physiopathologie des Maladies Psychiatriques, Institute of Psychiatry and Neuroscience of Paris, Institut national de la santé et de la recherche médicale (INSERM) 1266, Paris, France; ^3^ Department of Neurosurgery, Groupe Hospitalier Universitaire (GHU) Paris Psychiatrie et Neurosciences, Site Sainte-Anne, Paris, France; ^4^ Université Paris Cité, Institute of Psychiatry and Neuroscience of Paris (IPNP), Institut national de la santé et de la recherche médicale (INSERM) U1266, Paris, France; ^5^ Department of Psychiatry, Service Hospitalo-Universitaire, Groupe Hospitalier Universitaire (GHU) Paris Psychiatrie et Neurosciences, Site Sainte-Anne, Paris, France; ^6^ Cognitive Neuroimaging Unit, NeuroSpin Institut national de la santé et de la recherche médicale-Commissariat à l'énergie atomique et aux énergies alternatives (INSERM-CEA), Gif-sur-Yvette, France; ^7^ Institut de Neuromodulation, Groupe Hospitalier Universitaire (GHU) Paris, Psychiatrie et Neurosciences, Centre Hospitalier Sainte-Anne, Pôle Hospitalo-universitaire 15, Université Paris Cité, Paris, France; ^8^ Department of Neurosurgery, Perelman School of Medicine, University of Pennsylvania, Philadelphia, PA, United States; ^9^ Department of Surgery, Corporal Michael J. Crescenz Veterans Affairs Medical Center, Philadelphia, PA, United States; ^10^ Eating disorders Center, Clinique Villa Montsouris, Paris, France; ^11^ Department of Functional and Stereotactic Neurosurgery, Timone University Hospital, Marseille, France; ^12^ Aix Marseille Univ, Assistance Publique - Hôpitaux de Marseille (APHM), Institut national de la santé et de la recherche médicale (INSERM), Institut de Neurosciences des Systèmes (INS), Inst Neurosci Syst, Timone Hospital, Epileptology Department, Marseille, France; ^13^ Department of Neuroradiology, Groupe Hospitalier Universitaire (GHU) site Sainte-Anne, Paris, France

**Keywords:** feeding and eating disorders, treatment-resistant, electric stimulation therapy, ventral striatum, safety, intraoperative complications, postoperative complications, habits

## Abstract

**Background:**

Affecting adolescent and young adults, anorexia nervosa (AN) has the highest death rate of all mental disorders. Effective treatment options are lacking and a significant proportion of patients develop severe and chronic forms of the disease with long-lasting functional impairment. Neurobiology of AN implicates the nucleus accumbens as a core structure of the ventral striatum highly connected to the prefrontal cortex, the insula and the limbic system. Several studies reported promising results of deep brain stimulation for treatment-resistant AN. The aim of this study was to investigate the safety and efficacy of bilateral nucleus accumbens deep brain stimulation in severe and enduring AN.

**Methods and analysis:**

This is a prospective, multicentre, single-arm, open-label, non-randomized pilot trial of bilateral nucleus accumbens deep brain stimulation for severe and enduring AN. Patients will be followed up for 24 months after deep brain stimulation. The main objective of this study is to measure the safety and feasibility of nucleus accumbens deep brain stimulation in this population. The recruitment rate will be collected prospectively. Potential deep brain stimulation efficacy will be monitored by changes in: 1) health-related quality of life; 2) weight; 3) eating disorder symptomatology; 4) neuropsychological changes of cognitive flexibility, habits formation, emotional processing and central coherence; 5) psychiatric comorbidities (anxiety, depression, obsession). Local field potential recordings during an exposure task will be proposed to the patients. Additionally, caregiver quality of life will be assessed.

**Discussion:**

We present the design and rationale for a pilot study investigating the safety of nucleus accumbens deep brain stimulation for treatment resistant anorexia nervosa. This trial will provide an estimated effect size of nucleus accumbens deep brain stimulation for treatment-resistant anorexia nervosa to support future larger-scale clinical trials.

## 1 Introduction

### 1.1 Background and rationale

Anorexia nervosa (AN) is the psychiatric disorder with the highest mortality rate – the standardized mortality rate is higher than four – due to somatic complications and suicide (1–3). The median age at onset of AN is 15.5 years old (4). Despite the lack of longitudinal data and significant heterogeneity of care pathways, approximately 20% of patients develop a long-lasting disorder (5). In a staging approach, this subgroup of patients is considered to suffer from “severe and enduring AN” (SE-AN). Patients with SE-AN may benefit from specific treatments focusing on improving their social functioning and quality of life (6). At present, treatments for SE-AN are largely lacking (7).

The core position of the nucleus accumbens (NAc) is now well described in AN (8). NAc is involved in top-down control in decision-making through a fronto-accumbal hyperconnectivity (9), in food choice (10), and in body image disturbance through its connectivity with the insula (11). Numerous studies identified the mesocortico-limbic dopaminergic system in controlling eating behavior and body weight (12–15). Projections from the ventral tegmental area to the NAc are largely involved in the motivational aspect of food intake (16). Functional neuroimaging studies performed on acute and weight-recovered patients show that the NAc is a key structure associated with remission after refeeding (9, 17). The specific faculty to delay reward, as measured by the delay discounting task, involves the NAc (17). Preclinical studies identified an imbalance between ventral (NAc) and dorsal (caudate/putamen) striatum for the transition from reward-guided and goal-directed behaviors to automatic habitual behavior (18). These compulsive behaviors are common to both AN and obsessive-compulsive disorder (19).

Deep brain stimulation (DBS) of the NAc in mouse models of AN shows increase of food intake (20, 21) while, in a domestic pig model, DBS of the NAc shows modulation of the activity of the reward circuit structures (22). Recent series have reported promising results with NAc deep brain stimulation in patients suffering from severe and enduring AN (23–25).

Several promising reports concerning DBS in patients suffering from SE-AN have been published (23–31). In particular, the seminal studies by the group of Toronto showed that surgery and DBS are well tolerated in this population (28, 29). Nevertheless, these previous studies present some limitations. First, the DBS target was not systematically designed for AN. Second, included patients had frequently a very low body mass index (BMI). The BMI increase should not be the only studied endpoint since medical treatment alone cand lead to an acceptable BMI in AN (32–35). BMI is influenced by many variables other than pathophysiological improvement in the subpopulation of patients with SE-AN. Repetitive inpatient treatment can temporarily increase or stabilize BMI without quality of life improvement. While BMI at discharge remains a well-characterized prognostic factor (36, 37), recent studies have highlighted the need for a harm reduction-based treatment (38). Pilot studies assessing treatment acceptability and tolerability are necessary to explore the possibility of a subsequent larger, randomized, trial (39, 40).

### 1.2 Objectives

The main objective of the present study is to assess the feasibility and the safety of NAc DBS – during a single-stage surgery or two-staged surgery - in patients with SE-AN. Serious adverse events following DBS surgery will be monitored. The secondary objective is to measure the efficacy of NAc DBS. Clinical variables concerning recruitment rate, changes in BMI, changes in neurocognitive impairment, and changes in quality of life for the patient and their caregiver, will be prospectively collected. Local Field Potential (LFP) while performing provocation task will be recorded during two-staged surgery. This prospective pilot trial will help to build a larger randomized trial.

### 1.3 Trial design

We present the protocol for a multicentre, prospective, open-label, uncontrolled interventional trial, of bilateral NAc-DBS for SE-AN. The trial is currently open for recruitment at the GHU Paris Psychiatrie et Neurosciences Sainte Anne Hospital (Paris, France) and at the Clinique Villa Montsouris (Paris, France). These centers are specialized for the management of patients suffering from eating disorders abiding to the criteria of the French Federation Anorexia Bulimia and the General Directorate of Healthcare [Direction Général de l’Offre de Soins]: the teams from GHU Paris Psychiatrie et Neurosciences Sainte Anne Hospital and Clinique Villa Montsouris are working together (even before this trial) in the Ile-de-France TCA network (Réseau TCA Francilien; https://www.reseautca-idf.org/) and participate to the monthly multidisciplinary meeting attended by psychiatrists, psychologists, neurologists, neuroradiologists and neurosurgeons (https://www.ghu-paris.fr/fr/rcp-neuromodulation). In this way, prior to applying for grant funding, all referring physicians have given their consent to participate. They have received specific training by psychiatric and neurological teams of the GHU Paris Psychiatrie et Neurosciences already trained in deep brain stimulation programming.

## 2 Methods

### 2.1 Study setting

This is a multicentre, prospective, uncontrolled interventional trial with repeated pre- and per-stimulation measurements. The population of interest is patients suffering from SE-AN. After enrolment, patients will perform a complete preoperative workup, including metabolic assessment, neurocognitive evaluation, including emotional processing to specific AN stimulus, brain MRI. A DBS system will be implanted during surgery under general anesthesia. A 24-month follow-up will allow to observe and manage, if necessary, possible DBS-related complications and to perform two postoperative workups. DBS parameters will be optimized during this 24-month follow-up.

The sponsor of this study is the GHU Paris Psychiatry and Neurosciences, an academic hospital located in the fourteenth district of Paris (France). It is responsible for monitoring the study. It received a grant from the French Ministry of Health via the Programme Hospitalier de Recherche Clinique Interrégional 2019 (AOR19101-ZANELLO). No company is involved in funding this study.

### 2.2 Participants

The three different inclusion centers are all located in the Ile de France (Paris area) region. Two of them are university hospitals: 1) GHU Paris Psychiatry and Neuroscience Sainte Anne (Paris, France), 2) Paul Brousse Hospital (Public Assistance - Paris Hospitals, Paris, France), while the other one is a private clinic: 3) Clinique Villa Montsouris (CLINEA, Paris, France). They have a dedicated unit for eating disorders and are recognized as nationwide expert centers according to the French Federation Anorexia Bulimia (https://www.ffab.fr/).

Potential participants are reviewed by the investigators concerning inclusion and exclusion criteria (**Table 1**). The most important criterion is the definition of chronic and treatment-resistant anorexia nervosa (41, 42). This criterion is a composite criterion: 1) minimum duration of the anorexia nervosa of 7 years; 2) failure of at least two hospitalizations in a specialized center during the history of the disease, more precisely inability to maintain a BMI ≥ 17kg/m² for 2 months following hospitalization in a specialized center for the management of patients suffering from eating disorders (abiding to the criteria of the French Federation Anorexia Bulimia and the General Directorate of Healthcare [Direction Général de l’Offre de Soins]); 3) failure of at least two outpatient treatments conducted by a specialized team during the history of the disease (including at least one during the year preceding inclusion), in detail inability to maintain a BMI ≥ 17kg/m² for more than three consecutive months while following an outpatient treatment conducted by a specialized center for the management of patients suffering from eating disorders (abiding to the criteria of the French Federation Anorexia Bulimia and the General Directorate of Healthcare [Direction Général de l’Offre de Soins]). Eligible patients are discussed in a multidisciplinary meeting of the psychiatric, neurological, neuroradiological, and neurosurgical teams. Patients presenting both anorexia nervosa and bulimia nervosa are not excluded. There are no changes to the participants’ medical or psychotherapeutic/behavioral treatment(s) after enrolment before surgery. Any changes to treatments following surgery are documented, including dosages and indications for change.

**Table 1:** Study inclusion and exclusion criteria.

Inclusion Criteria
1. Presence of Anorexia nervosa according to DSM V criteria. 2. Age 18 to 65 years. 3. Chronic, treatment-resistant anorexia nervosa, defined as:
- Anorexia nervosa evolving for at least 7 years - Inability to maintain a BMI ≥ 17kg/m² for 2 months following hospitalization in a specialized center for the management of patients suffering from eating disorders (abiding to the criteria of the French Federation Anorexia Bulimia and the General Directorate of Healthcare [Direction Général de l’Offre de Soins]). Failure of at least 2 hospitalizations in a specialized center during the history of the disease. - Inability to maintain a BMI ≥ 17kg/m² for more than 3 consecutive months during the year preceding the inclusion, while following an outpatient treatment conducted by a specialized center for the management of patients suffering from eating disorders (abiding to the criteria of the French Federation Anorexia Bulimia and the General Directorate of Healthcare [Direction Général de l’Offre de Soins]). Failure of at least 2 outpatient treatments conducted by a specialized team during the history of the disease
4. Impaired psychological, social, and occupational functioning defined by a score ≤ 45 on the Global Assessment of Functioning Scale. 5. Anorexia nervosa is judged to be the primary disorder if there are psychiatric comorbidities such as depression, anxiety disorder, obsessive-compulsive disorder, or personality disorder, by at least two independent experts. 6. The patient is able to comply with the operational and administrative requirements of the study and is able to complete the protocol questionnaires. 7. Patient provides written informed consent. 8. Patient is drug-free or on a medication that has been stable for at least 6 weeks at the time of the study entry. 9. If female subject and of childbearing age: use of an effective method of contraception. 10. Patient has health insurance.
Exclusion Criteria
1. Presence of a concurrent major clinical disorder (Axis I disorder) that is primary toanorexia nervosa confirmed by both a clinical interview and a structured interview. 2. Presence of a personality disorder assessed by structured interview that could compromise compliance with post-surgical follow-up according to 2 independent psychiatrists 3. Presence of severe neurological pathology or significant MRI abnormalities (excluding anorexia-related atrophy). 4. Cognitive and intellectual capacity to understand the risks and constraints of the technique or to give informed consent. 5. Albumin levels <30g/L. 6. Presence of medical contraindications to undergo implantation of a deep brain stimulation system or to receive an MRI (non-MRI compatible pacemaker for instance). 7. Pregnant or breastfeeding woman. 8. History of deep brain stimulation for anorexia nervosa or other pathology. 9. Contraindication to general anesthesia. 10. Presence of multiple comorbidites or active generalized infections. 11. Patient deprived of liberty by judicial or administrative decision. 12. Patient under forced psychiatric care. 13. Patient admitted to a health or social institution for purposes other than research. 14. Minor patient (under 18 years of age). 15. Patient under legal protection (guardianship, curatorship). 16. Patient unable to give consent.

### 2.3 Intervention: DBS surgery

The experimental medical device is a DBS device: Infinity™ system. It is composed of two DBS directional electrodes (6172), extensions (60 cm if possible to ensure the MRI compatibility, or 90 cm if not possible), and the non-rechargeable implantable neurostimulator. The manufacturer is the ABBOTT Laboratories (Abbott Park, IL, USA). The treatment, i.e. high-frequency electrical stimulation, is then delivered continuously and over the long term. The hospitalization in neurosurgery lasts seven days.

The different stages of the DBS surgical procedure at GHU Paris Psychiatrie et Neurosciences Sainte Anne are:

- The patients undergo a preoperative MRI scan. This MRI is performed on a 3-T Vantage Galan ZGO scanner (Canon Medical Systems Corporation, Otawara, Japan). The sequences are the following: 1/three-dimensional (3D) T1-weighted fast spoiled gradient-recalled acquisition; 2/two-dimensional (2D) coronal accelerated fast spin echo scan T2-weighted focused on nucleus accumbens; 3/Spin Echo DTI 50 directions; 4/3D Susceptibility-weighted angiography sequence; 5/3D CUBE Fluid attenuated Inversion recovery; 6/3D CUBE T2-weighted; 7/contrast material–enhanced three-dimensional (3D) T1-weighted fast spoiled gradient-recalled acquisition (gadoterate meglumine [Dotarem; Guerbet, Aulnay-sous-Bois, France], 0.1 mmol/kg).
- Surgery consists of fitting two DBS electrodes, the extensions (cables linking the electrodes to the neurostimulator) and the neurostimulator. The surgery (in one stage or in two stages) takes place under general anesthesia. It is performed with a robotic arm (Neuromate, Renishaw) and an intraoperative 3D imaging device (O-Arm 2.0, Medtronic). Depending on patient agreement, this surgery may be carried out in one stage or in two stages.

The one-stage surgery consists of a unique procedure during which the electrodes are implanted and connected to the extensions, which in turn are connected to the neurostimulator.

During the two-stage surgery, the leads are connected to externalized temporary extensions first. The patient is then discharged from the operating theatre and LFP are recorded (see below). After 4 days, the patient returns to the operating room, the temporary extensions are removed and the electrodes are connected to the permanent extensions, which in turn are connected to the neurostimulator.

C. Post-operative monitoring systematically involves post-operative imaging and biological sampling in addition to the clinical checks (healing follow-up, neurological examination, evolution of the symptoms of the SE-AN). On day 1 after the lead implantation, a postoperative biological sampling and a CT scan (LightSpeed; GE Healthcare, Milwaukee, Wisconsin, USA) without contrast enhancement are performed to check the correct positioning of the electrodes and the absence of postoperative biological and radiological complications. A second follow-up CT scan may be discussed, given the possible movement of the directional stimulation electrodes at a distance from the surgery. This second CT head scan may therefore be carried out three months after the electrodes have been implanted, to ensure that the positioning of the directional contacts has not changed.

During the first postoperative week, neurostimulation is started on the following parameters: 2 mA – 90µseconds – 130 Hertz using the adapted tool (Physician programming system, Apple™ Ipad Mini, ABBOTT). At day 7, i.e. at the theoretical discharge of the patient, the patient’s notebook and patient card are handed over, and the following neurostimulation parameters (4 mA – 90 µseconds – 130 Hertz using the adapted tool) are set.

The eventual subsequent changes in the stimulation parameters are left to the discretion of the research team and are validated during a multidisciplinary meeting. In this way, programming the DBS system is performed by a member of the research team during a visit in addition to the usual check-ups (see below). The medical treatment can be changed during the stimulation period: all modification is recorded into a dedicated eCRF (see below).

### 2.4 Outcomes

#### 2.4.1 Primary endpoint

The primary endpoint is the proportion of serious adverse reactions (SAR) occurring in the patients included in the study. For continuous safety monitoring, we adopt a binomial sequential test, re-evaluated with each new patient, with the following parameters: maximum proportion of patients with SAR 15%, bilateral alpha 15%, beta 20%. The maximum and minimum tolerance limits for SAR were determined using the toxbdry function of the clinfun package version 1.0.15 of R. Taking the lower limit as a precaution, **Table 2** summarizes the maximum number of patients with a tolerable SAR to respect a threshold below 15%. This study design is justified because complications directly related to surgery typically appear within the first month (43). There are long-term complications of DBS which are taken into account in the protocol as the patients are followed up (44). These long-term complications must be put into perspective with the risk of death associated with SE-AN (2). The first three patients will be included sequentially with an interval of at least one month to ensure that there are no systematic adverse effects. If the 15% threshold of SAR in the study population is exceeded, as assessed in a sequential procedure, the study will be stopped.

**Table 2:** Maximum number of patients with a serious adverse event to respect a threshold below 15%.

Number of included patients	Maximum limit of the interval
3	1
4	1
5	1
6	1
7	1
8	2
9	2
10	2
11	2
12	3

Highlighted numbers correspond to the maximum tolerated threshold of patient(s) presenting a serious adverse reaction related to the surgery (rounded down).

Serious adverse events are defined by the French National Agency for the Safety of Medicines and Health Products (ANSM) based on the French Public Health Code: “A serious adverse event is an adverse event that is lethal, or likely to be life-threatening, or leading to significant or lasting disability or incapacity, or causing or prolonging hospitalization, or manifesting itself as a congenital anomaly or malformation.”

SAR are serious adverse events directly caused by the intervention. In particular, we look for:

1. Intracerebral hematoma directly related to surgery resulting in permanent neurological deficit,
2. Cerebromeningeal infection directly related to surgery resulting in a life-threatening condition during treatment,
3. Development of drug-resistant epilepsy directly related to the surgery and affecting the patient’s quality of life,
4. Permanent neurological deficit(s) induced by the activation of the brain stimulation,
5. Major psychiatric impairment directly related to the surgery and endangering the patient’s vital prognosis.

Four different levels of causality are used: 1) Not related; 2) Possible; 3) Probable; and 4) Causal relationship. The intensity of the adverse events is assessed using the Common Terminology Criteria for Adverse Events (Version 5.0, Published: November 27, 2017).

Concerning any eventual SAR, the causality will be discussed during a meeting with the Independent Monitoring Committee. For instance, suicide or suicide attempts could be or not be directly related to the surgery (45). Indeed, Parkinson’s disease patients operated with deep brain stimulation demonstrated a lower risk of suicide relative to Parkinson’s disease patients without deep brain stimulation (46). At first, any serious adverse event (such as a suicide) will result in the protocol being stopped until the cause of this possible SAR is characterized.

#### 2.4.2 Secondary endpoints

Secondary endpoints are a measurement of DBS efficacy and acceptability.

##### 2.4.2.1 DBS efficacy

**Figure 1:**
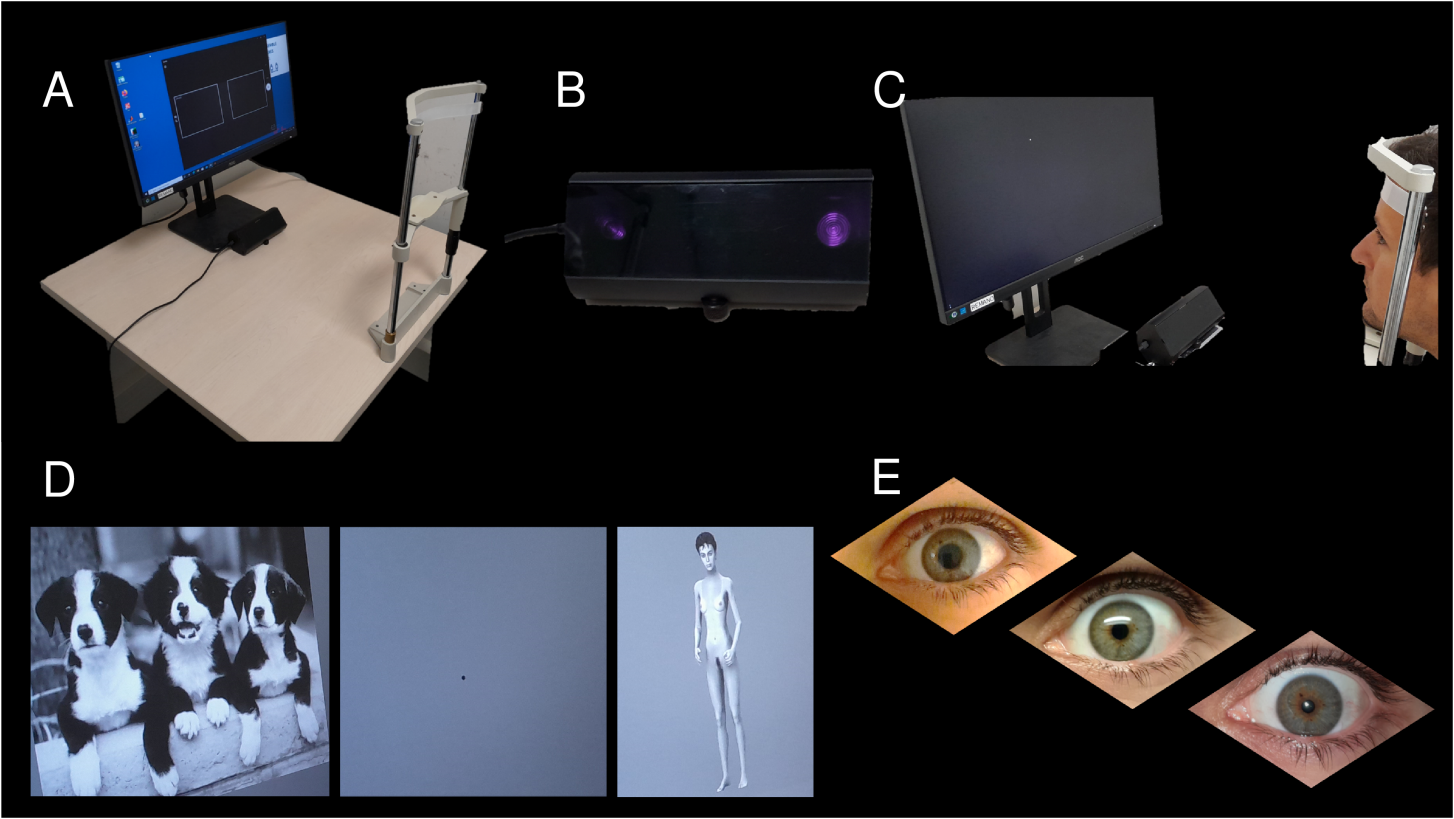
Details of the pupillometry. **(A)** the pupillometry set-up with a chin rest, a computer with a 21″ screen, and an eye tracker; **(B)** the eye tracker (LiveTrack Fixation Monitor, Cambridge Research Systems Ltd, UK); **(C)** the first step is to calibrate the camera for pupil tracking during test; **(D)** different image sets are proposed in random order (neutral images, violent images, sexual images, different silhouette images) with a washout image (a black spot, see middle image) between each test image; **(E)** different pupil sizes. Pupillometry was performed in a windowless room with low luminosity.

For the measurement of DBS efficacy, we rely on:

1. Duration of hospital stays in a specialized center. Comparison between the two years before inclusion in the STIMARS protocol and the two years after inclusion (with a 6-month washout interval right after the DBS surgery to consider stimulation start and initial parameter adjustments).
2. BMI monitoring in a standardized way. Monthly measurements during the follow-up according to the calculation rule: weight (kilograms)/height² (square meter) during each follow-up visit with the referring physician. Comparison between the two years before inclusion and the two years after inclusion (with a 6-month interval to consider stimulation start and first parameter adjustments). Additionally, body composition will be assessed by multifrequency impedancemetry Z-Métrix^®^ (Bioparhom). Resting energy expenditure will be measured by indirect calorimetry with Omnia 1.6.10 - Quark RMR (COSMED).
3. Changes in preoperative and postoperative scores on the various clinical dimensions of AN via various questionnaires (see **Table 3**). Eleven dimensions of eating disorders will be measured by the *Eating Disorder Inventory-2* (EDI-2) (47). Levels of anxiety and depression will be assessed through the *Hospital Anxiety and Depression* Scale (48). The presence and severity of obsessive-compulsive disorder will be measured through the *Yale-Brown Obsessive Compulsive Scale* (Y-BOCS) (49, 50). These data will be collected during the preoperative, early postoperative, and late postoperative neuropsychological assessments (3 months, 12 months, and 23 months after inclusion, respectively). Additionally, the Zarit scale aims to evaluate the burden of the patient’s severe and resistant anorexia nervosa on a caregiver: the caregiver present at the inclusion visit must be 1) present during the patient’s neuropsychological assessments or 2) available by telephone to complete the scale (duration of the interview: 15 minutes, including 5 minutes for completing the scale).
4. Changes in neurocognitive dimensions. All cognitive tasks will be performed on computers (with MATLAB^®^) under the supervision of a trained neuropsychologist.
  - Changes in pupillometry assessments (**Figure 1**). Pupillometry measures the pupil diameter for an objective evaluation of the emotional impact of a task on the subject. The pupil dilates in response to positive and negative emotions, as well as in response to light. Using a subtractive method, the effect of light is removed and the emotional impact is quantified. We use pupillometry (dedicated eye tracker, chin rest, and a dedicated computer; see **Figure 1**) to comparatively assess the emotional impact caused by 60 computer-viewed pictures belonging to different types of stimuli: 12 neutral images (e.g. image of a book), 12 negative images (e.g. a violent image), 12 pleasant images (e.g. image of babies, kittens), 6 images of caloric foods (e.g. image of ice cream), 6 images of lean food (e.g. image of carrots), and 12 images of standardized silhouettes depicting different BMI. Emotional stimuli (neutral, pleasant, and negative images) were taken from the International Affective Picture System (IAPS) (51). Food pictures come from the Open Library of Affective Foods (52). Standardized silhouettes were computer-generated pictures (53), representing a naked woman (height 165 cm) varying in BMI, with underweight silhouettes (BMI: 12–16 kg/m2), normal weight silhouettes (BMI: 19–23 kg/m2) and overweight silhouettes (BMI: 26–30 kg/m2). All pictures are in grey levels, 8 bits, 1024x768px, modified with TheGIMP^©^. The task was programmed using MATLAB^®^. Stimuli were randomized into 12 blocks. Each block containing five pictures: three IAPS pictures (one positive, one negative, one neutral), one food picture, and one standardized silhouette. Each picture will be shown for 6s with a 2s in‐between fixation point. The quantitative aspect of the emotional impact is given by the changes in pupil diameter, unlike the sign of the emotion (positive or negative) which requires an additional questionnaire: at the end of the task, subjects will view the pictures a second time and will be asked to evaluate valence and emotional arousal, using a simple numeric scale. Questions will be ‘Would you rate this image as pleasant or unpleasant?’ from 1 (very unpleasant) to 9 (very pleasant), and ‘What is the emotional impact, how powerful is the feeling caused by this picture?’ from 1 (very low) to 9 (very high).
  - Time specificities (capacity to delay reward) will be measured through the Delay Discounting Task (DDT). In the DDT, subjects are presented with two options of various amounts of money available at different delays: one smaller amount of money but available within a shorter delay (“Smaller Sooner”, SS) and one larger amount of money but available in a longer delay (“Larger Later”, LL). This task allows measuring the subjective value that a person perceives in a given reward depending on the delay.
  - Cognitive flexibility will be measured through the Brixton test (54, 55), the Trail-Making Test (56) and the Wisconsin Card Sorting Test (57). Previous research suggests that this test has no practice effect (58).
  - The Slip-of-action Task (SOAT) (59, 60) will assess reward-motivated versus habit-driven behavioral choices. The task is detailed in a previous publication (18) and measures the tendency to lose sight of a goal (goal/reward-motivated choices) due to the activation of habitual responses (habit-driven choices).

**Table 3:** Details of neurocognitive assessment.

Anorexia Nervosa Dedicated Form
1. the Eating Disorder Inventory 2
Psychiatric Forms
1. Hospital Anxiety and Depression Scale 2. Yale Brown Obsessive-Compulsive Scale 3. Scale for Suicide Ideation
Neurocognitive Forms
1. Montreal Cognitive Assessment 2. Delay Discounting Task 3. Wisconsin Card Ranking Test 4. Brixton Test 5. Trail Making Test A and B 6. Slip-of-action test 7. Habits vs Goal Directed Behavior test 8. Dubois Five Word Test 9. Rey/Taylor Figure
Quality of life Forms
1. SF-36 Quality of Life Questionnaire 2. Global Assessment of Functioning Scale
Care-taker Form
1. Zarit Burden Inventory (to be completed by the care-taker)

###### 2.4.2.1.1 DBS acceptability

In this study, the DBS acceptability of the method is measured via simple objective numerical measures (percentage of patients who accepted inclusion, percentage of patients who completed the study, percentage of patients willing to repeat the DBS procedure if necessary) and more complex criteria which are sought during follow-up consultations (61). The criteria sought during follow-up consultations are those described in the Theoretical framework of acceptability: affective attitude, burden, ethicality, intervention coherence, opportunity costs, perceived effectiveness, and self-efficacy (62).

##### 2.4.2.2 Local field potential recording during an exposure task

**Figure 2:**
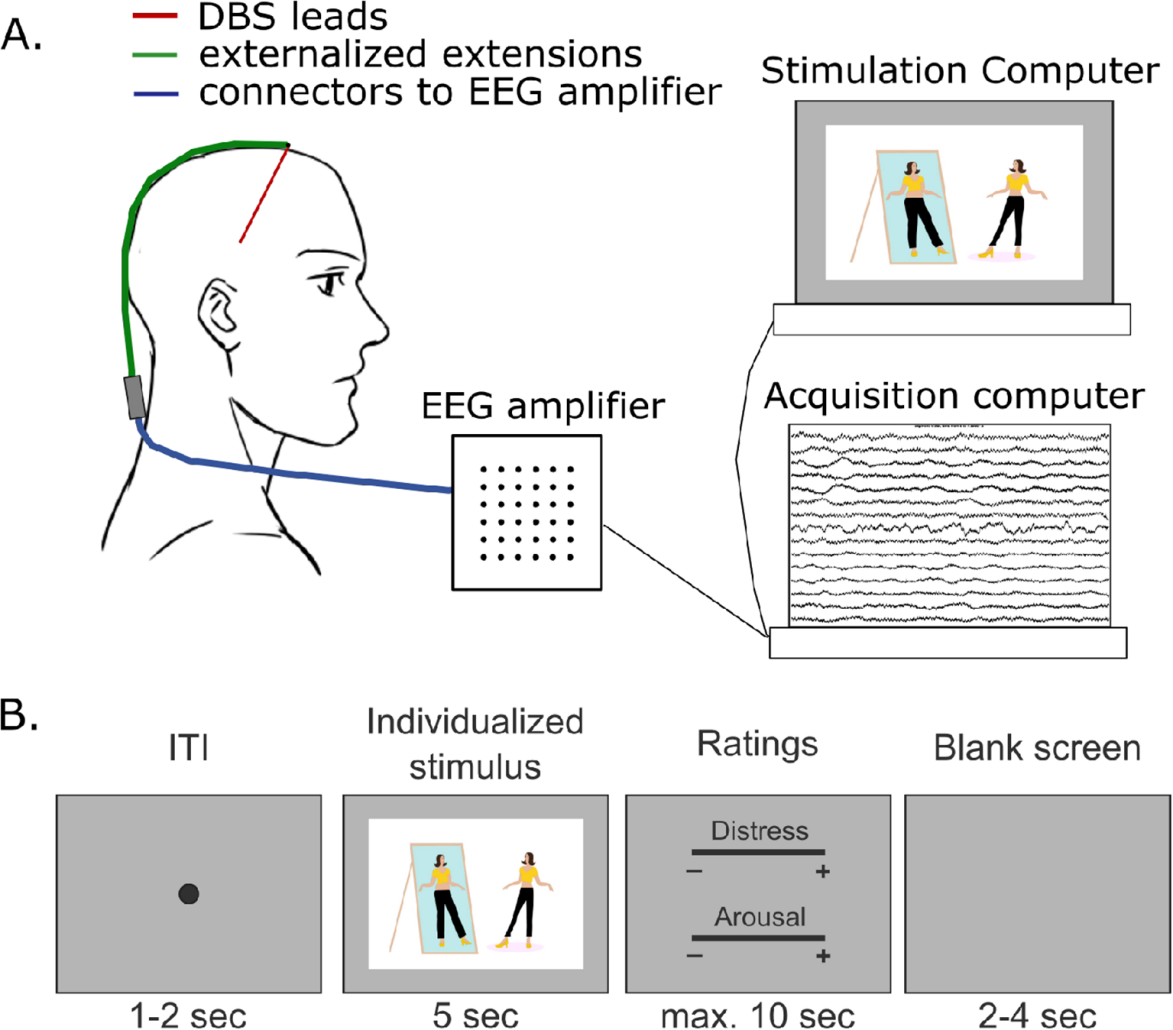
Details of the local field potential recording during an exposure task. **(A)** the leads are externalized after the surgery. Two days after the surgery, the extensions are connected to an electroencephalogram amplifier, and then to two computers, one for the signal analysis and the other one for the exposure task. **(B)** the exposure task consists in presenting 120 stimuli. From left to right: each stimulus will be composed of a fixation point (random time from 1 to 2 sec), followed by the image (5 sec), followed by two scales on which the patients will have to rate the induced level of distress and the arousal of the image (visual analog scales from 0 to 5, random order of scale presentation, maximum time for the ratings of 10 sec), followed by a blank screen (random time from 2 to 4 sec).

**Figure 2** summarizes NAc LFP recording.

We optionally propose patients perform LFP recordings in the NAc during an exposure task, based on individualized symptoms-related stimuli. If a patient agrees to perform this procedure, the surgery is two-staged (see above).

###### 2.4.2.2.1 Stimuli

During the exposure task, four types of stimuli are used: individualized- and generic- symptoms-related, and neutral pictures. Individualized stimuli are obtained by asking the patient to take pictures in his or her environment during the month before surgery. Generic stimuli, selected from a dedicated database, are added to increase the number of trials and the signal-to-noise ratio. Symptoms-related pictures are typically scales, body images, and sweet/high-calorie foods while neutral pictures are random objects or landscapes. All stimuli are normalized for luminance and contrast.

###### 2.4.2.2.2 Task

Pictures are presented in blocks of 15 trials. Each trial is composed of a fixation point (random time from 1 to 2 sec), followed by the image (5 sec), followed by two scales on which the patient has to rate the induced level of distress and the arousal of the image (visual analog scales from 0 to 5, random order of scale presentation, maximum time for the ratings of 10 sec), followed by a blank screen (random time from 2 to 4 sec). In total, 120 stimuli are presented during the task.

###### 2.4.2.2.3 Procedure

LFP will be recorded from the DBS macroelectrodes targeting the NAc the second day after the lead implantation and for two days. The day after surgery is dedicated to the patient’ recovery. The exposure task is performed using a 15-inch screen for the display, and a button pad for the ratings, while the patient is sitting on a chair. The patient is free to rest as long as needed in between blocks of trials, and to stop the experiment at any time if distress or discomfort is too high.

###### 2.4.2.2.4 Recordings

LFP are recorded bilaterally using definitive leads (directional leads 6172 ABBOTT), which have 8 platinum-iridium contacts: one full ring contact, then two contacts with equally spaced three segments then one last full ring contact. Contact dimensions are 1.29 mm in diameter, 1.5 mm in length, and contact spacing 0.5 mm. The signal is amplified and digitized at 2048 Hz using an electroencephalogram amplifier (Refa system; TMSi, Oldenzaal, Netherlands).

###### 2.4.2.2.5 Analyses

Bipolar recordings are reconstructed by filtering from 1 to 200Hz and subtracting signals between adjacent contacts of the electrodes. An artifact detection procedure is applied to reject a portion of a signal contaminated by electrical or movement-related noise. Bipolar signals are then segmented in trials and the time-frequency maps are computed using the adaptive multitaper algorithm. The statistical differences in power between symptoms-related and neutral images are assessed using linear models, including the induced level of distress, the arousal, and the familiarity of the images as factors. Signal processing, visualization, and statistical analyses are performed using MNE-python and R software (63). The analysis of LFP recordings during the exposure task are used at the population level to assess the presence of symptoms-related activity in the NAc and its frequency signature. These recordings are also eventually used at the individual level to localize pathological activities within the NAc to identify the best parameters for the stimulation such as the stimulating contacts.

### 2.5 Participant timeline

A detailed schedule for trial participants is presented in **Table 4**.

**Table 4:** Detailed schedule for trial participants.

Assessment	*Visit(s) with the referring physician* (prior to the trial)	Eligibility Visit 1 -4 weeks	Enrollment Visit 2 Week 0	Preoperative Visit 3 weeks after Visit 2	Surgery 4 weeks after Visit 3	Postoperative Visits 5-29					End of trial Visit 30 24 months after surgery (+/- 1 week)	*Post-trial* At least every 3 months
						Every month (+/- 1 week)	Every 2 months (+/- 1 week)	3 months after surgery (+/- 1 week)	12 months after surgery (+/- 1 week)	23 months after surgery (+/- 1 week)		
Eligibility screen	**X**	**X**										
Informed consent for trial			**X**									
Care-taker informed consent for trial			**X**									
Informed surgical consent				**X**								
Neurosurgical examination			**X**	**X**	**X**		**X**					**X**
Psychiatric examination		**X**	**X**		**X**	**X**						**X**
Body Mass Index measurement		**X**	**X**	**X**	**X**	**X**	**X**	**X**	**X**	**X**	**X**	**X**
Serious Adverse Events monitoring				**X**	**X**	**X**	**X**	**X**	**X**	**X**	**X**	**X**
Anesthetic evaluation				**X**								
Biological tests				**X**	**X**							
MRI				**X**								
Deep brain stimulation surgery					**X**							
Postoperative CT					**X**							
The Eating Disorder Inventory 2				**X**				**X**	**X**	**X**		
Psychiatric Scales												
1. Hospital Anxiety and Depression Scale 2. Yale Brown Obsessive-Compulsive Scale 3. Scale for Suicide Ideation				**X**				**X**	**X**	**X**		
Neurocognitive Scales												
1. Montreal Cognitive Assessment 2. Delay Discounting Task 3. Wisconsin Card Ranking Test 4. Brixton Test 5. Trail Making Test A and B 6. Slip-of-action test 7. Habits vs Goal Directed Behavior test 8. Dubois Five Word Test 9. Rey/Taylor Figure				**X**				**X**	**X**	**X**		
Quality of life Scales												
1. SF-36 Quality of Life Questionnaire 2. Global Assessment of Functioning Scale				**X**				**X**	**X**	**X**		
Care-taker Scale												
1. Zarit Burden Inventory (to be completed by the care-taker)				**X**				**X**	**X**	**X**		
Pupillometry				**X**				**X**	**X**	**X**		

Patients are informed by their referring practitioner of the existence of the protocol and the surgical possibilities before the selection visit (so outside the trial itself). Patients eligible for inclusion are discussed at a multidisciplinary meeting attended by psychiatrists, psychologists, neurologists, neuroradiologists, and neurosurgeons. Referring practitioners work in reference centers for the management of anorexia nervosa: patients deemed clinically or psychologically unstable are not presented at the multidisciplinary consultation meeting. Only after a positive collegial opinion has been obtained from the multidisciplinary consultation meeting can patients undergo the inclusion visit.

Patients who meet the inclusion criteria and do not have exclusion criteria are informed about the protocol during the selection visit. The inclusion visit takes place at least 2 weeks after the protocol description: this is a mixed consultation necessarily associating a psychiatrist specialized in the management of eating disorders and a neurosurgeon specialized in DBS. The patient’s trusted person and/or his relatives is present during this consultation. During the inclusion visit, the patient is asked to give or not his consent to participate in the study. The necessary steps before a DBS surgery (anesthesia assessment, MRI, blood tests) and the first neurocognitive assessment are performed during the preoperative visit. The patient has at least three visits and 8 weeks between the selection visit and the surgery.

The duration of follow-up after patient inclusion is 24 months. The patient is followed up at least monthly by the referring physician. These visits are part of the patient’s routine care: their frequency can therefore be modulated according to the care required but can never be less than monthly. The minimal investigations conducted during these consultations are as follows: 1) structured psychiatric interview; 2) measurement of BMI; 3) collection of SAR; 4) verification of the functioning (impedance measurement in particular) of the neurostimulation using the appropriate equipment (Physician programming system, Apple™ Ipad Mini, ABBOTT).

Concurrently, the patient will attend bi-monthly consultations with the neurosurgeon. Specific investigations performed during these consultations include: 1) a complete clinical examination including general physical examination, detailed examination of scars by the neurosurgeon; 2) verification of the proper functioning of the neurostimulation.

DBS programming is performed during a mixed consultation involving a psychiatrist specializing in the management of eating disorders and a neurosurgeon specializing in DBS. This mixed consultation can replace a simple neurosurgical consultation.

The included subjects benefit from three postoperative neurocognitive assessments: an early one, which takes place 3 months after surgery, a late one, which takes place 12 months after surgery, and a final one, which takes place 23 months after surgery. These assessments repeat the same tests as those performed during the preoperative visit.

Patients can keep the neurostimulator at the end of the study if they wish. The team that implanted the neurostimulator, the GHU Paris Psychiatry and Neuroscience team, manages it at the end of the protocol. The GHU Paris Psychiatry and Neuroscience hospital bears the costs. It should be noted that patients can then benefit from the implantation of a rechargeable internal pulse generator, which reduces costs.

### 2.6 Data collection and management

An electronic case report form (eCRF) is used (Redcap^©^ v10.6.11, Vanderbilt University). Data entry is performed by the study investigators and their collaborators on this eCRF, on password-protected computers. Data are collected at each follow-up consultation and each neurocognitive assessment (the preoperative one and the 3 postoperative). The data collected directly in the CRFs and considered as source data are notably summaries of structured interviews with the patient, summaries of physical examinations, including measurements of the BMI, and collections of SAR within the follow-up booklet by the patient and at each follow-up consultation.

Participants consent in writing to the statistical treatment and scientific publication of these data in strict anonymity. Consequently, they will only be identified by their identification code. The lead investigator will conserve a participant identification list that will be used only if records need to be identified. Files will be stored on a password-protected computer in accordance with local data protection law and will be handled in strict confidence.

Documents related to the study will be conserved for the legal duration of 15 years and archives will be kept in a locked room under the responsibility of the lead investigator. Data cannot be moved or destroyed without the authorization of the lead investigator.

### 2.7 Security and adverse events

#### 2.7.1 Data monitoring

A clinical research associate (CRA) appointed by the sponsor, the GHU Paris Psychiatry and Neuroscience, ensures that the research is carried out correctly, and that the data generated are documented, recorded, and reported, by the Standard Operating Procedures implemented within the Direction de la Recherche Clinique et de l’Innovation from the GHU Paris Psychiatrie et Neurosciences Sainte Anne and in compliance with Good Clinical Practice and the legislative and regulatory provisions in force. Quality control visits are conducted monthly by the CRA.

#### 2.7.2 Adverse events

Adverse events are at the heart of this study since the primary endpoint is the proportion of SAR occurring in patients. Participants will be asked about SAR at every study visit. Each serious adverse event will be reported by the principal investigator to the study promotor. These serious adverse events will be monitored until their resolution. They may lead to discontinuation of the protocol.

### 2.8 Statistical methods

#### 2.8.1 Sample size

This is a pilot study and is not intended to evaluate the device’s effectiveness. The required number of participants was set at 12, following the Julious’s recommendations (64). Considering that seven patients out of sixteen presented serious adverse event(s) in one of the largest reported series to date, 12 patients should be enough to detect this effect size (28, 65).

#### 2.8.2 Statistical analysis

Statistical analysis will be performed at the end of the follow-up of all patients. The statistical analysis will be made in the biometrics center of the GHU Paris Psychiatry and Neurosciences. Data will be analyzed using JMP software (Version 14.1.0; SAS Institute Inc, Cary, North Carolina, USA). All analyses will be realized with a two-sided type I error rate of 5%. Quantitative data will be described by means and medians, standard deviations and interquartile ranges. Nominal data will be depicted by percentages with two-sided 95% confidence intervals. Time-to-event (time-to-relapse) data will be plotted by the Kaplan-Meier method. The proportions of missing data will be calculated. Patient characteristics (eligibility criteria, demographics, history, data at inclusion) will be described and compared by center. Quantitative data will be compared using Student’s t-test for a single sample (paired t-test) or ANOVA for repeated measures or non-parametric tests (Wilcoxon in paired series) when the distribution remains skewed even after appropriate transformation (Tukey power scale). Categorical data (pre vs. post) will be compared using Chi-2 or Fisher’s exact tests and or McNemar’s test for repeated measures.

### 2.9 Ethics and dissemination

The study protocol was approved by the Comite de Protection des Personnes (CPP) Est-II (n°: 21.05.12.69252) and by the ANSM (French National Agency for Medicines and Health Products Safety). Investigators will ask all participants to give written informed consent before participation, and all data will be recorded anonymously. The study will be conducted according to ethics recommendations from the Helsinki Declaration (World Medical Association, 2013).

The need to repeatedly inform patients and ensure their understanding of the implications of this protocol was taken into consideration when drafting this protocol (66–68). Several measures have been taken to guarantee fair, clear, and appropriate information: 1) patient cases are systematically discussed during multidisciplinary meetings; 2) patients are only referred by physicians who have been treating them for years (referring physicians); 3) delays of several weeks are always respected between each step of the protocol, 4) the implication of a caregiver is mandatory for inclusion. An Independent Monitoring Committee (IMC) checks patient recruitment and the continuation in the study. The first meeting of the IMC has to be done before the first inclusion and then the IMC meets at least twice annually to discuss possible safety issues. The four members of the IMC are independent of sponsor, laboratory, and investigator. The members of the IMC are: one biostatistician with expertise in clinical research; two neurosurgeons with expertise in functional neurosurgery and DBS; one psychiatrist with expertise in the management of SE-AN. The existence of psychiatric emergencies within the GHU Paris Psychiatry and Neuroscience hospital and the long history of links between psychiatry and neurology guarantee high standards in terms of research ethics (69, 70).

All data collected during this trial are the property of the research sponsor, the GHU Paris Psychiatry and Neuroscience, and may not be communicated to a third party under any circumstances without the written consent of the sponsor. At the end of the research, the participant has the right to be informed of the research’s overall results, according to the modalities that will be specified during the inclusion interview. Participants will have the possibility of communicating their e-mail addresses to the investigator who collected their consent to obtain the overall results of this research when they become available.

The results will be published after final analysis in scientific articles in peer-reviewed journals and may be presented at national and international conferences. Any publication or communication (oral or written) is decided by mutual agreement between the investigators and the sponsor. Individuals who contribute to the production of the publication will be granted authorship.

## 3 Discussion

Patients suffering from treatment-resistant AN have limited treatment options nowadays. They exhibit poor outcomes, even in expert centers (71). Our study protocol aims to support the growing literature on DBS for treatment-resistant AN.

Costs associated with AN are mostly related to hospital stays (72). The average length of stay for patients with AN is higher than 100 days in Europe (73). During hospitalization in expert centers, refeeding approaches lead to a clinical remission according to the BMI, even with higher-calory refeeding (74). However, relapse of AN arises frequently during the 18 months after hospitalization (75). This underlines the need to manage more community-oriented therapeutic approaches in AN patients. Our study does not require very low BMI to include the patient: the aim is more to manage the deinstitutionalization of AN patients than to obtain weight gain.

Whereas the safety of DBS is now well-established in neurological conditions, this question remains for treatment-resistant AN patients (76, 77). This study will help to refine the expected complication rate in front of these malnourished patients. Moreover, DBS is reversible and customizable. These two points explain why DBS, a reversible neuromodulation technique, should always be considered rather than irreversible lesion techniques (such as thermolesions, radiosurgery) in psychiatric patients.

The main limitations of this study are the single arm and open-label characteristics. This study design has been considered as mandatory by the French authorities. After confirmation of the DBS safety in patients with treatment-resistant AN, a larger study will be possible. The small study sample is in line with the main outcome of safety. We have decided to monitor the patient’s quality of life with two scales translated and validated in French, to minimize the common misgrading of quality of life in anorexia nervosa patients (78). However, these scales are generic and an eating disorder-specific measure would represent a nice to further studies (79). The measurement of healthcare intervention acceptability should investigate multiple aspects: despite numerous psychiatric follow-up visits, only limited data will be collected during the protocol (62). The development of a specific theory-informed questionnaire about DBS for SE-AN would have been a significant improvement and should be done for the next studies (80, 81).

The strengths of this study are the use of a directional DBS system, the target chosen for AN purpose only, and the patient inclusion not during a severe relapse of their treatment-resistant AN. This will help to obtain the real effect size of NAc DBS for treatment-resistant AN.
